# Evaluation of an online case study-based course in translational science for a broad scientific audience: Impacts on students’ knowledge, attitudes, planned scientific activities, and career goals

**DOI:** 10.1017/cts.2022.415

**Published:** 2022-06-07

**Authors:** Amanda L. Vogel, Shadab F. Hussain, Jessica M. Faupel-Badger

**Affiliations:** Education Branch; Office of Policy, Communications and Education; National Center for Advancing Translational Sciences; National Institutes of Health; Bethesda, MD, USA

**Keywords:** Translational research, translational science, education, training, workforce, curriculum

## Abstract

**Purpose:**

There is a need for education activities in translational science (TS) that focus on teaching key principles, concepts, and approaches to effectively overcome common scientific and operational bottlenecks in the translational process. Delivering this content to the broad range of individuals interested in advancing translation will help to both expand and develop the TS workforce. Rigorous evaluations will build the evidence base for effective educational approaches for varied audiences.

**Methods:**

In 2020, the National Center for Advancing Translational Sciences offered an online case study-based course in TS for students across education and career stages. The course evaluation used baseline and endpoint student surveys to assess satisfaction with the course and impacts of participation on knowledge and attitudes relevant to TS and professional goals.

**Results:**

Of 112 students, 100 completed baseline and/or endpoint surveys, with 66 completing both. Most found the online format (*n* = 59, 83%) and case study approach (*n* = 62, 87%) moderately or very effective. There were statistically significant increases in TS knowledge (*P* < .001) and positive attitudes about team science in translational research (TR) (*P* < .001). Students reported the course increased their skills and knowledge in cross-disciplinary team science, the process of preclinical and clinical TR, and how their work fits into the translational spectrum, and increased their interest in scientific approaches used in the case study and careers in TS, TR, or team science.

**Conclusions:**

This online case study-based course effectively conveyed TS concepts to students from a range of backgrounds and enhanced their professional interests related to course content.

## Introduction

Translational science (TS) education aims to convey core concepts in TS – including effective scientific and operational approaches and strategies – that have been shown to effectively prevent or resolve common scientific and operational bottlenecks that slow or stall the translational process. In doing so, it aims to equip current and future members of the biomedical research workforce with the knowledge and skills they need to advance research along the translational spectrum. Examples of TS core content include as follows: approaches for enhancing research efficiency to accelerate translational timelines; methods for stimulating creativity and innovation in a research initiative; strategies for identifying unmet research and health needs and pursuing paradigm changing research goals; methods for collaborating effectively in cross-disciplinary teams; and approaches to generate and maintain effective partnerships across government, industry, and academia; among many others [1].

As investments in TS continue to grow, there are increasing opportunities to become involved via scientific, administrative, and partnership activities. This is reflected in the growing number and diversity of individuals showing interest in obtaining training and education in TS [2, 3, 4]. These individuals are coming to the field with a range of academic and professional backgrounds, spanning training and career stages, and with varied professional goals.

To adequately respond to both the demand for a larger TS workforce and the varied characteristics of those wishing to participate, there is a need to expand the range of education opportunities in TS to create additional openings into the field. Currently, most opportunities for TS training and education occur within defined predoctoral or postdoctoral training programs at leading academic medical centers [2, 3]. These in-depth training and education opportunities are essential to build a cadre of translational researchers with skills for advancing their research along the translational spectrum. Additional educational opportunities are needed, as well, to reach those earlier in their training (i.e., undergraduates), mid-career investigators, and the range of individuals involved in managing, facilitating, supporting, and partnering in translation (e.g., project managers, team science specialists, funders, and patient advocates). New educational opportunities will need to employ teaching approaches tailored to these varied audiences. Moreover, these new offerings will need to be scalable to reach the larger number of students this represents.

It will also be critically important to rigorously evaluate new TS education opportunities to build the evidence base for effective educational approaches for diverse scientific audiences. Evaluations should assess the effectiveness of new education opportunities to transmit core TS concepts by using objective measures of change in students’ knowledge. They should also assess longer-term outcomes, such as the impact of participation on students’ professional goals and activities. Such evaluations also should look for differences in course effectiveness and impacts based on students’ educational backgrounds, career stages, professional roles and work sectors, and learning goals. Doing so will help to identify effective educational approaches for a broad range of interested participants.

In 2020, the Education Branch of the National Center for Advancing Translational Sciences (NCATS), National Institutes of Health (NIH), piloted a short course in TS concepts including scientific and operational principles for success and examples of how these principles are implemented in practice. The course was created as a proof of concept to test the effectiveness of an online format and case study-based teaching approach to convey TS concepts to a broad scientific audience [5].

The course was accompanied by a rigorous evaluation involving baseline and endpoint student surveys. The evaluation assessed students’ satisfaction with the course design and impacts of participation on students’ knowledge and attitudes relevant to TS, planned scientific activities, and career goals. It also analyzed key outcomes by students’ backgrounds with relevance to the course content. Here, we briefly describe the course – which is described in greater detail in a separate publication [5] – and provide a detailed summary of the evaluation design and findings. We end with a discussion of the implications of these findings for developing, implementing, and evaluating future education opportunities in TS.

## Methods

### Course Design and Recruitment of Students

“MEDI 501: Principles of Preclinical Translational Science: A Case Study from Cancer Drug Discovery and Development” was a 7-week one-credit online course designed by the NCATS Education Branch and offered in both summer and fall 2020 through a partnership with the Foundation for Advanced Education in the Sciences (FAES) located at the NIH [5].

Multiple strategies were used to reduce barriers to participation. There were no prerequisites for enrollment, and the course was advertised broadly. This included postings to professional society listservs (e.g., Association of American Medical Colleges), NIH listservs and email lists (e.g., NIH Science of Team Science listserv), and the NCATS and FAES websites. The postings reached NCATS stakeholders and extramurally funded scientists and trainees, internal NIH audiences, and the broader scientific community (e.g., foundations and patient advocacy groups). The course registration fee, paid to FAES, was significantly subsidized by NCATS, resulting in a nominal registration fee of only $50 per person. Registration was on a first-come, first-served basis, and both the summer and fall 2020 sessions of this course reached capacity within days of registration opening. A total of 112 individuals enrolled over the two terms.

The online format was selected for its scalability and potential to enhance accessibility to TS education. Toward maximizing access, the course was offered almost entirely asynchronously to account for students with varied schedules and across time zones. During each week of the course, students viewed prerecorded lectures, read required and recommended readings, and completed assignments at their own pace. The course included two live question and answer sessions with course faculty. Students were able to submit questions ahead of time and sessions were recorded for later viewing for students who could not attend in real time.

The case study-based teaching approach was selected to convey complex content to a broad scientific audience. For this pilot course, the case featured a successful preclinical research project conducted by NCATS in collaboration with the National Cancer Institute (NCI), Northwestern University, and the University of Kansas that led to development of a highly promising drug candidate to treat advanced metastatic cancer, called metarrestin, which is currently in phase 1 clinical trials [6].

This particular case was selected because it demonstrated core concepts and effective approaches in TS and leveraged in-house experts to teach the case. The project focused on an unmet scientific and patient need – the lack of a specific treatment targeting the metastatic process in cancer, which is the primary cause of cancer deaths [7]. It pursued a novel scientific and clinical concept in cancer metastasis to develop a first-in-class compound to treat metastasis. The project team pursued this goal through highly innovative approaches including a clinical biomarker-based phenotypic approach, rather than a target-based approach specific to the metastatic process, that ultimately led to the identification of a novel target for the treatment of metastasis [8]. In addition, the case demonstrated the essential roles of cross-disciplinary team science, cross-agency collaborations, and an organizational environment that encourages both, to advance the science [9].

This case study offered the advantage that it could be taught by NCATS staff members who were leaders of the many scientific teams that contributed to the project, including biologists, chemists, toxicologists, and pharmacologists. These faculty members described the science they conducted and offered their first-person perspectives on scientific and operational principles of TS that were demonstrated in this project, how these principles were implemented, their benefits for advancing the science, and their generalizability to other initiatives.

Additional details about the course are provided in a companion article, which also includes the course syllabus and other teaching materials, as well as initial findings about student characteristics and satisfaction with the course design [5]. More information also can be found on the NCATS Education Branch webpages at https://ncats.nih.gov/training-education/resources.

### Evaluation Design

The course evaluation design was informed by the Kirkpatrick Evaluation Model, which identifies four levels of outcomes and impacts for educational offerings: (1) satisfaction with the course; (2) knowledge acquisition; (3) behavioral and attitudinal change; and (4) impact on performance [10].

Data collection consisted of baseline and endpoint student surveys administered in the first and last weeks of the course. The survey instruments included both quantitative and qualitative questions that collected information on student characteristics including: background relevant to the course; learning goals; degree of participation in the course; satisfaction with the course, particularly the online format and case study-based teaching approach; change in TS knowledge and attitudes from the start to end of the course, and variation in these outcomes with students’ backgrounds relevant to the course content; and impact of participation on students’ skills, attitudes, and plans for future scientific activities and professional goals. In addition, the endpoint survey collected students’ thoughts about the value of the course to them, personally, and provided the opportunity to recommend ways to improve the course and content to add if the course were expanded to a two-credit offering. The survey instruments are provided in the Supplementary Materials.

To assess change in students’ knowledge and attitudes related to course content, a scale with each focus area was developed and included in both the baseline and endpoint survey instruments. The knowledge scale focused on assessing students’ knowledge of TS approaches in preclinical and clinical drug discovery and development research. It included 17 items soliciting self-report assessment of current knowledge of 5 topics: general concepts in TS, drug discovery approaches, drug development approaches, clinical trials approaches, and collaborations and partnerships. Students rated their current knowledge of each item on a five-point Likert scale from “no knowledge” to “expert knowledge.”

The attitudinal scale focused on attitudes about collaboration in translational research (TR). It included 10 items assessing attitudes about three topics: team science in TR, cross-disciplinary team science in TR, and ability to learn skills and knowledge for cross-disciplinary team-based TR. Students indicated how strongly they agreed or disagreed with each statement with responses on a five-point Likert scale from “strongly disagree” to “strongly agree.” This new scale was adapted from the Research Orientation Scale, which assesses attitudes about cross-disciplinary team science in general [11]. While not formally validated and tested, both new scales were developed with input from the full course faculty. Together, they offered a wide range of relevant feedback rooted in their related expertise, which contributed to refining the scales.

The evaluation tested five hypotheses, as follows:

H1: Overall, there will be a statistically significant positive increase in scores on both the (a) knowledge and (b) attitudes scales.

H2: Students with fewer years of experience in TR will have greater increases in scores on both the (a) knowledge and (b) attitudes scales.

H3: Students with and without a background in cancer biology will have similar increases in scores on the knowledge scale.

H4: Students with no prior background in drug discovery and development will have a greater increase in scores on the knowledge scale compared to students with this background.

H5: Students with lower baseline scores on the knowledge scale will have greater increases in scores on both the (a) knowledge and (b) attitudes scales.

Quantitative data analyses were conducted in SAS, and included frequencies, paired t-tests, and independent samples t-tests. The t-tests were used to compare mean change in knowledge and attitudes among different groups of students. Qualitative data analyses comprised thematic analysis of text responses to open-ended questions. Excel was used to support these analyses. This evaluation research was approved by the NIH Institutional Review Board (project number P205038).

## Results

### Sampling Frame and Sample

A total of 112 students participated in the 2 course sessions, and 100 responded to either the baseline or endpoint survey. This included 95 students who completed the baseline survey, of whom 66 also completed the endpoint survey, and 5 who completed the endpoint survey only. Of the 66 students who completed both surveys, 62 provided valid responses to the TS knowledge and attitudes scales. The other four students’ responses to the endpoint scales did not pass attention checks. These responses were discarded and excluded from the paired t-test analyses. However, these individuals’ other endpoint survey responses were retained because they showed traits of high-quality data including internal validity.

### Respondent Characteristics

Students varied in both their training and career stages and their educational backgrounds. More than half had a doctoral degree (60%), while a quarter had a bachelor’s degree. Students reported over 30 different disciplines for their highest degrees, with biology (30%) most frequently reported, followed by medicine, chemistry, and biochemistry. More than a third (38%) of students were participating more than 5 years after receipt of their highest degree. Just over half (59%) had a background in cancer biology and about half (48%) had a background in drug discovery and development. Two-thirds (68%) reported that their current work contributes to TR. Students reported a variety of work settings, with about two-thirds in academia (67%) (Table 1).

**Table 1.** Student characteristics at baseline (n = 95)

	No. (%)
Highest degree	
PhD	48 (51)
Bachelors	24 (25)
Masters	13 (14)
MD	6 (6)
MD/PhD	3 (3)
Other	1 (1)
Disciplinary training, by highest degree	
Biology	29 (30)
Medicine	12 (13)
Chemistry	9 (9)
Biochemistry	8 (8)
Other	37 (39)
Years since highest degree	
0–5 years	58 (62)
6–10 years	19 (20)
11–15 years	9 (10)
More than 15 years	8 (8)
Current student	
Yes	26 (28)
Background in Cancer Biology	
I have been involved in conducting cancer biology research	32 (33)
I have been involved in conducting other cancer research	31 (32)
I have academic training in cancer biology	29 (30)
I have been involved in providing cancer patient care	10 (10)
None of the above	39 (41)
Background in Drug Discovery and Development (D&D)	
I have been involved in conducting drug D&D research	35 (36)
I have academic training in drug D&D	23 (24)
I have been involved in business, administrative, or legal work around drug D&D	10 (10)
None of the above	50 (52)
Translational research (TR) experience	
Less than 1 year	38 (40)
1–2 years	24 (25)
3–5 years	19 (20)
6–10 years	13 (14)
More than 10 years	1 (1)
Current work contributes to TR	
Yes	65 (68)
Currently teach a course in skills for TR	
Yes	4 (4)
Work sector	
Academia	64 (67)
Government	23 (24)
Industry/business/private sector	3 (3)
Nonprofit/NGO	4 (4)
Other	1 (1)

### Respondents’ Learning Goals and Satisfaction with the Course

Students had a range of learning goals for the course, reflecting interest in acquiring practical knowledge and skills and introductory-level education in both TS and drug discovery and development. There was high student participation in the course, as reflected in viewing course lectures and completing required readings. In addition, students were highly satisfied with the course. Most students reported that the online format and case study approach were moderately or very effective to teach the course content (93% and 87%, respectively). Nearly, all reported that the course moderately or completely achieved its aim to “provide a unique window into the TS process.” Finally, nearly all reported that the course was moderately or extremely valuable to them (Table 2).

**Table 2.** Learning goals, participation, and satisfaction with the course

Baseline evaluation questions (*n* = 95)	No. (%)
Learning goals^*^	
Obtain knowledge and skills that I can apply in my future career	71 (74)
Obtain knowledge and skills that I can apply in my current work	57 (59)
Get an introduction to translational science	54 (56)
Get an introduction to drug discovery and development	43 (45)
Learn how others are teaching translational research skills, to help me develop/enhance my own course on this topic	27 (28)
Other	4 (4)

Endpoint evaluation questions (*n* = 71)	No. (%)
Degree of participation in the course^**^	
Viewed 51%–100% of lectures	67 (94)
Completed 51%–100% of required readings	53 (75)
Completed 51%–100% of recommended readings	17 (24)
Online format effectiveness	
Somewhat effective	12 (17)
Moderately effective	18 (25)
Very effective	41 (58)
Case study approach effectiveness	
Somewhat effective	9 (13)
Moderately effective	12 (17)
Very effective	50 (70)
Degree to which course achieved its aim to provide a unique window into translational science	
Slightly	1 (1)
Moderately	22 (31)
Completely	48 (68)
Overall value of course to the respondent	
Slightly valuable	5 (7)
Moderately value	23 (32)
Extremely valuable	43 (61)

* Students selected one or more learning goals.

** Categories not mutually exclusive.

### Respondents’ Change in Knowledge and Attitudes

**Fig. 1.**
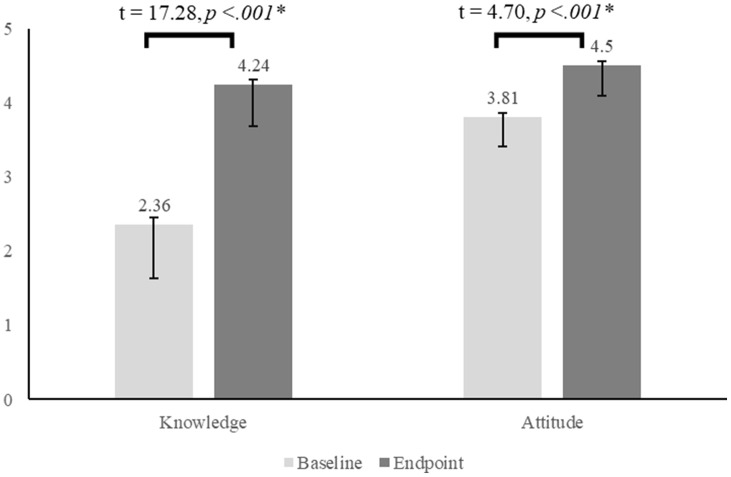
Mean change in students’ knowledge and attitudes, results of paired sample t-tests. Note: Error bars show the standard error of the mean.

Fig. 1 shows results related to Hypothesis 1, which posited that, overall, there would be a statistically significant positive increase in scores on both the: (a) knowledge and (b) attitudes scales among all students. Fig. 1 shows that the results supported this hypothesis.

Table 3 presents data related to Hypotheses 2 through 5, which investigated variations in increases on the knowledge and attitudes scales based on students’ relevant pre-course experience, backgrounds, and knowledge. Compared to students with more years of TR experience, students with fewer years of TR experience had a significantly greater increase in scores on the knowledge scale (H2a), but not on the attitudes scale (H2b). Students with and without a background in cancer biology had similar increases in scores on the knowledge scale (H3). Compared to students with a background in drug discovery and development, students with no prior background in this topic had a significantly greater increase in scores on the knowledge scale (H4). Compared to students with higher baseline scores on the knowledge scale, students with lower baseline scores on this scale had significantly greater increases in scores on both the knowledge and attitudes scales (H5a and H5b).

**Table 3.** Change in knowledge and attitudes by students’ backgrounds, baseline knowledge, and course engagement (n = 62)

Hypotheses	Group 1 (*n*)		Group 2 (*n*)		t-Value	CI	*P*-value
	M	SD	M	SD			
	≤2 years of TR experience (*n* = 41)		≥3 years of TR experience (*n* = 21)				
2a. Years of TR experience (K)	1.58	.65	1.19	.62	2.34	[.06, .74]	.02^*^
2b. Years of TR experience (A)	.27	.46	.22	.34	.39	[-.18, .27]	.70

	No reported background (*n* = 27)		Some reported background (*n* = 35)				
3. Cancer biology background (K)	1.46	.74	1.45	.60	.10	[-.33,.36]	.92

	No reported background (*n* = 37)		Some reported background (*n* = 25)				
4. Drug discovery background (K)	1.59	.58	1.24	.72	2.11	[.02, .69]	.04^*^

	Baseline score: 1–2 (*n* = 25)		Baseline score: 3–5 (*n* = 37)				
5a. Baseline TS knowledge (K)	1.87	.56	1.17	.57	4.80	[.41, .99]	<.001^*^
5b. Baseline TS knowledge (A)	.40	.46	.15	.03	2.39	[.04, .46]	.02^*^

K = outcome is preclinical translational science knowledge scale score; A = outcome is team science in translational research attitudes scale score; TR = translational research; TS = translational science.

* t-Test result is statistically significant at α = .05

### Course Impacts on TS Skills, Knowledge, Planned Scientific Activities, and Career Goals

The endpoint survey included optional open-ended questions that invited students to report on how the course influenced their goals for their future work and how the course influenced the approaches they intend to use in their future work. It also included an optional open-ended question that invited students to explain their rating for the value of the course to them, overall.

Out of the 71 endpoint survey respondents, 41 individuals (57.7%) responded to 1 or more of these 3 questions. These students reported that the course enhanced their skills and knowledge in four key areas: (1) skills and knowledge for cross-disciplinary team-based research; (2) knowledge of what TS is, and its value to enhance TR; (3) knowledge of the process of preclinical and clinical TR; and (4) understanding of how their own work fits into the translational spectrum. Students also reported that the course influenced their plans for future scientific activities and career goals in three areas: (1) it created or reinforced a desire to focus one’s career on TS, TR, and/or team science; (2) it increased their interest in drug discovery and development approaches highlighted in the case study; and (3) it increased positive attitudes about and interest in participating in cross-disciplinary team-based TR. Table 4 offers exemplar quotes that illustrate these themes.

**Table 4.** Course impacts on skills, knowledge, planned scientific activities, and career goals

Theme	Exemplar quotation
Influence on skills and knowledge	
Skills and knowledge for cross-disciplinary team-based research	“I fully intend to create a visual model of a project at the beginning of collaboration with a new team. I thought that was a great idea to get everyone on the same page and is especially helpful for visual learners.”
Knowledge of what translational science (TS) is and its value to enhance translational research (TR)	“I really had little to no knowledge of what translational science was, nor what it could achieve. Now I see that it is a highly effective tool that can make translational research much more efficient.”
Knowledge of the process of preclinical and clinical TR	“Getting to know how different disciplines collaborate and what objectives/roles each part is involved in gave me a better understanding of therapeutic development.”
Understanding of how their own work fits into the translational spectrum	“I will be more aware of all the steps involved in pre-clinical research when I interact with patient organizations and researchers.”
Influence on planned scientific activities and career goals	
Created or reinforced a desire to focus one’s career on TS, TR, and/or team science	“Participating made me want to pursue translational research as the main focus of my research career, long-term.”
Increased their interest in drug discovery and development approaches highlighted in the case study	“I am now more curious to experiment more with phenotypic screening and similar experiments to solve the problems I care about, rather than focusing entirely on single gene products.”
Increased positive attitudes about and interest in participating in cross-disciplinary team-based TR	“This course influenced my drug development ideas for future studies. By utilizing pharmaceutical chemists as possible future collaborators, I think it could accelerate our drug discovery methods.”

### Recommendations

Two optional open-ended survey questions invited students to recommend ways to enhance the course or expand it to a two-credit offering. A total of 48 students (67.6% of endpoint respondents) completed questions that solicited this feedback. To enhance course content, students recommended adding more depth on principles of TS, team science, and preclinical research innovations. Students recommended adding new topics for an expanded course, including patient engagement, dissemination of findings to stakeholders, clinical trials and their relationship to preclinical research, filing of investigational new drug (IND) applications with the US Food and Drug Administration (FDA), and manufacturing. Students also expressed an interest in learning from multiple case studies. Finally, students recommended making highly technical scientific lectures more accessible to students with limited or no related scientific background by reducing technical language and improving quality of closed captioning and making self-pacing more flexible.

## Discussion

### Summary of Purpose and Findings

New and innovative educational opportunities will be essential to prepare the growing number of individuals who are interested in contributing to advancing translation. Effective education opportunities will need to be tailored to students from diverse disciplinary backgrounds, across training and career stages, and with varied goals for their contributions to advancing translation, including research, administration, funding, partnerships, and other contributions.

In response to this need, the NCATS Education Branch piloted an online case study-based course to convey complex TS concepts to a broad scientific audience and conducted a rigorous course evaluation. Findings offered evidence for the effectiveness of the course to convey TS content to students with a range of backgrounds and to positively influence their planned scientific activities and career goals. That said, students with less prior experience and knowledge in the course content had greater knowledge gain from the course. These findings suggest that future courses designed at different levels – including introductory and higher level – might be beneficial. Likewise, student requests to make technical lectures more accessible highlight the potential benefits of courses designed with different audiences in mind.

Students’ written responses about the impacts of the course on their TS skills and knowledge, plans for future scientific activities, and career goals highlighted the practical nature of the course. They also demonstrated that the impact of the course went beyond knowledge gain to influence their scientific behaviors and goals, which are the ultimate targets of TS training and education.

### Implications for Future TS Education Opportunities

Given the positive findings from this course evaluation, the NCATS Education Branch has developed additional TS courses that use the same design principles, including the online format, case study-based approach, and faculty roles for both internal experts in the case and experts in core principles of TS. These courses are also open to a broad scientific audience. While the MEDI 501 course focuses on preclinical research, subsequent courses together cover all stages of the translational spectrum [12]. These include a course that teaches TS core concepts through multiple short case studies of COVID-19 research across the translational spectrum (specifically, preclinical and clinical research, implementation science, and public health research), and a course that teaches TS core concepts through the lens of rare disease research, which similarly highlights TS approaches that span the translational spectrum [12].

Student recommendations on the MEDI 501 course have already been used for quality improvements to MEDI 501 and to inform planning for subsequent courses. In 2021, the MEDI 501 course was enhanced to include additional lectures and readings on the topics of TS principles, team science in the metarrestin team, and approaches to stimulate creativity and innovation in TR. Other recommendations that were not specific to the MEDI 501 course pointed to students’ interest in TS along the translational spectrum, including specific interests in patient engagement, dissemination of findings, IND filing and interactions with the FDA, and manufacturing and scale-up, as well as interest in learning from additional case studies. These recommendations are being taken into consideration as content is being developed for future educational offerings from the NCATS Education Branch.

Different institutions have different levels of resources where course development and evaluation are concerned. The case study-based approach has the advantage of drawing upon an institution’s past research successes and internal experts to generate and deliver course content. It is therefore a highly adaptable approach that could be taken up more broadly. In addition, the case study-based teaching approach also can be right-sized based on the resources available and leveraged for a range of teaching or training modalities. For example, written narratives of case studies can be used for discussion sessions, and lectures about cases could be provided in more compressed formats such as seminars. In addition, while a single in-depth case can be developed to teach many core concepts and approaches in TS, brief cases can be used to focus in on particular concepts or approaches and may require fewer resources to construct.

Asynchronous online courses with prerecorded lectures are also a flexible approach for settings with varied resources. Zoom and similar platforms have made recording lectures a low-cost endeavor, and these courses can then be offered repeatedly with minimal effort, reaching large numbers of students with few additional investments over and above up-front investments (e.g., minimal annual updates based on student feedback or progress in the case at the center of the course). For additional guidance on teaching case study-based courses in TS, please see our companion articles on development and implementation of the MEDI 501 course [5] and proposed core content for TS education [1].

The course described here is one of a range of activities that the NCATS Education Branch has piloted toward broadening TS education and training opportunities. Other activities include intramural fellowships and internships in TS for postbaccalaureate, predoctoral, and postdoctoral participants and TS workshops [4, 5, 9, 13, 14]. In 2022, the Branch will be launching a summer internship program in TS with a specific focus on engaging trainees from backgrounds that have been historically underrepresented in the biomedical research workforce. Also toward increasing diversity in the TS workforce, subsequent to the courses described here, the branch began targeted advertising of our courses and training opportunities to academic departments with relevance to TS located at minority serving institutions (MSIs). The Education Branch is also considering ways to offer course content outside of the formal course structure, to enhance access.

### Implications for Evaluations of Future TS Education Opportunities

To develop evidence-informed practices in TS education, it will be critically important to rigorously evaluate current and future TS educational offerings and rapidly disseminate course designs and evaluation methods, instruments, and findings. This article joins a small body of published evidence of the effectiveness of case study-based teaching in TS (cf. Greenberg-Worisek, *et al*. [15]; Greenberg-Worisek, *et al.* [16]). We encourage others to adapt the evaluation instruments included in this article, as useful.

Future evaluations will benefit from being structured around conceptual models and educational objectives, including quantitative indicators of course impact, and objectively measuring course outcomes and impacts via administration of paired baseline and endpoint student surveys.

As the NCATS Education Branch continues to refine our conceptualization of TS core content and implement additional courses that focus on TS in other stages of the translational spectrum, we have recognized the need for a TS knowledge scale that is relevant across the full translational spectrum. In response, we have recently developed a scale that assesses knowledge of generalizable principles of TS. In future evaluations of the course described here, and in evaluations of other NCATS Education Branch case-based courses, this new scale will be used alongside a knowledge scale specific to the case or cases featured in the course to capture change in both areas of knowledge.

Another area for growth is testing and validation of scales capturing TS knowledge and attitudes. Future work by the NCATS Education Branch will speak to this need. Contributions in this area by the broad TS education and training community will provide valuable resources to advance evaluation in our field.

Given the critical importance of diversifying the TS workforce, another area of development in our evaluation activities is assessment of demographic diversity among course participants. In future evaluations, the NCATS Education Branch plans to collect student data including gender, race/ethnicity, and geographic location.

As the course described here was quite brief, this evaluation was designed to be low burden for participants. As a self-report scale, the knowledge scale was lower burden than an objective scale. It also offered multiple advantages for a course of this nature. One was the ability to use the scale to assess knowledge gain among a group of students with wide-ranging baseline knowledge of the course content. Another was the ability to capture change in broad domains of knowledge, which would be overly burdensome to capture in an objective scale. The course, itself, did include a weekly quiz to assess knowledge retention from the week’s lectures, which was not used as part of the evaluation.

It was beyond the scope of the evaluation to recontact participants to assess longer-term impacts of the course. To assess these impacts to some degree, this evaluation included questions about the impact of the course on goals and intentions for future professional activities, leveraging long-standing proxy measures for behavior change [17]. Longer-term education and training opportunities, such as certificate or degree programs and fellowship and internship opportunities, offer the potential to follow participants long term to assess distal impacts on scientific behaviors and goals, which are the ultimate targets of such education and training.

## Conclusions

Novel TS education opportunities that create new openings into the TS field will aid in engaging the growing number of individuals, with diverse experiential and educational backgrounds, who are interested in contributing to the field of TS. Moreover, they will equip participants with the skills to enhance the translational process, contribute to our TS knowledge base, and educate others in TS.

This evaluation of a pilot course in TS for a broad scientific audience provides evidence for the effectiveness of the online format and case study-based teaching approach to convey complex TS concepts to this audience and influence their planned scientific activities and career goals. We anticipate additional novel approaches in TS education from colleagues in the TS community and encourage rigorous evaluations and rapid dissemination of findings to build the evidence base for effective practices in TS education for the broad biomedical research workforce.

## Supplementary material

For supplementary material accompanying this paper visit https://doi.org/10.1017/cts.2022.415". click here to view supplementary material

## References

[1] Faupel-Badger JM , Vogel AL , Austin CP , Rutter J . Advancing translational science education. Clinical and Translational Science. Submitted. 10.1111/cts.13390 PMC9652430 36045637

[2] Sancheznieto F , Sorkness CA , Attia J , et al. Clinical and translational science award T32/TL1 training programs: program goals and mentorship practices. Journal of Clinical and Translational Science 2021; 6(1): e13. 35211339 10.1017/cts.2021.884 PMC8826009

[3] Sorkness CA , Scholl L , Fair AM , Umans JG . KL2 mentored career development programs at clinical and translational science award hubs: practices and outcomes. Journal of Clinical and Translational Science 2019; 4(1): 43–52. 32257410 10.1017/cts.2019.424 PMC7103475

[4] Haynes B , Brimacombe K , Hare C , Faupel-Badger J . The National Center for Advancing Translational Sciences’ intramural training program and fellow career outcomes. CBE Life Sciences Education 2020; 19(4): ar51. 33001768 10.1187/cbe.20-03-0048 PMC8693946

[5] Faupel-Badger JM , Vogel AL , Hussain SF , et al. Teaching principles of translational science to a broad scientific audience using a case study approach: A pilot course from the National Center for Advancing Translational Sciences. Journal of Clinical and Translational Science 2022; 6(1): e66. 10.1017/cts.2022.374 PMC9201875 35754433

[6] Frankowski KJ , Wang C , Patnaik S , et al. Metarrestin, a perinucleolar compartment inhibitor, effectively suppresses metastasis. Science Translational Medicine 2018; 10(441): eaap8307. 29769289 10.1126/scitranslmed.aap8307 PMC6176865

[7] Steeg PS. Targeting metastasis. Nature Reviews Cancer 2016; 16(4): 201–218. 27009393 10.1038/nrc.2016.25 PMC7055530

[8] Norton JT , Titus SA , Dexter D , Austin CP , Zheng W , Huang S . Automated high-content screening for compounds that disassemble the perinucleolar compartment. Journal of Biomolecular Screening 2009; 14(9): 1045–1053. 19762548 10.1177/1087057109343120 PMC2857721

[9] Vogel AL , Knebel AR , Faupel-Badger J , Portilla LM , Simeonov A . A systems approach to enable effective team science from the internal research program of the National Center for Advancing Translational Sciences. Journal of Clinical and Translational Sciences 2021; 5(1): e163. 10.1017/cts.2021.811 PMC8427549 34527302

[10] Kirkpatrick DL , Kirkpatrick JD. Evaluating Training Programs: The Four Levels. Oakland, CA: Berrett-Koehler Publishers, 2006.

[11] Hall KL , Stokols D , Moser RP , et al. The collaboration readiness of transdisciplinary research teams and centers: findings from the National Cancer Institute’s TREC year-one evaluation study. American Journal of Preventive Medicine 2008; 35(2 Suppl): S161–S172. 18619396 10.1016/j.amepre.2008.03.035 PMC3292855

[12] National Center for Advancing Translational Sciences. *Translational science training and education resources* [Internet], 2021. (https://ncats.nih.gov/training-education/resources)

[13] National Center for Advancing Translational Sciences. *Intramural research training opportunities* [Internet], 2021. (https://ncats.nih.gov/training-education/training/NCATS-opportunities)

[14] National Center for Advancing Translational Sciences. *Translational science interagency fellowship* [Internet], 2021. (https://ncats.nih.gov/training-education/training/TSIF)

[15] Greenberg-Worisek AJ , Campbell KA , Klee EW , et al. Case-based learning in translational biomedical research education: providing realistic and adaptive skills for early-career scientists. Academic Medicine 2019; 94(2): 213–216. 30256254 10.1097/ACM.0000000000002470 PMC6351155

[16] Greenberg-Worisek AJ , Cornelius KE , Cumba Garcia L , Enders FT , Shah ND , Windebank AJ . Translating innovation in biomedical research: design and delivery of a competency-based regulatory science course. Journal of Clinical and Translational Sciences 2019; 4(1): 8–15. 10.1017/cts.2019.432 PMC7103473 32257405

[17] Ajzen I. The theory of planned behavior. Organizational Behavior and Human Decision Processes 1991; 50: 179–211.

