# Supplementary material for: Evaluation of an online case study-based course in translational science for a broad scientific audience: Impacts on students’ knowledge, attitudes, planned scientific activities, and career goals
Source: J Clin Transl Sci. 2022 Jun 7;6(1):e82. doi: 10.1017/cts.2022.415 (PMC9305082; doi:10.1017/cts.2022.415)
Supplement: Supplementary file 1 [file S2059866122004150sup001.docx]

This document includes supplementary materials for the manuscript**:** Evaluation of an Online Case Study-Based Course in Translational Science for a Broad Scientific Audience: Impacts on Students’ Knowledge, Attitudes, Planned Scientific Activities, and Career Goals

This includes the baseline survey instrument and endpoint survey instrument used to evaluate the NCATS-developed course, “Principles of Preclinical Translational Science.”

We encourage readers to adapt relevant portions of these instruments for evaluations of their own courses, with proper citation of this publication.

**BASELINE SURVEY INSTRUMENT**

**Title: Help us Enhance the Course, “Principles of Preclinical Translational Science”: Pre-Course Survey**

| **Q#** | **Survey Question** |
| --- | --- |
| **1** | **Do you wish to participate in the survey?** (required question)  Yes, I consent to participate (skip pattern: go to question 2)  No, I decline to participate (skip pattern: go to closing screen) |
| **Please answer the following questions about your participation in this course.** | |
| **2** | **How did you hear about this course?** (e.g., FAES catalogue, particular listserv or website) ________________________________________________________________ |
| **3** | **Which of the following best reflects your learning goals for your participation in this course? (Select all that apply)**  Get an introduction to translational science  Get an introduction to drug discovery and development  Obtain knowledge and skills that I can apply in my current work  Obtain knowledge and skills that I can apply in my future work  Learn how others are teaching translational research skills, to help me develop/enhance a course on this topic  Other: _______________________________________________________________________ |
| **We are interested in learning about course participants’ background related to the course content. Please answer the following related questions.** | |
| **4** | **How much translational research experience have you had, to date?** (required question)  Less than 1 year  1 -2 years  3 -5 years  6-10 years  More than ten years |
| **5** | **Does your current work contribute to translational research? (e.g., laboratory, clinical, population-level, administrative, or training activities related to translational research)** (required question)  Yes  No  N/A – I’m a student |
| **6** | **Do you currently teach a course in skills for translational research?** (required question)  Yes (go to question 7)  No (go to question 8) |
| **7** | (Skip pattern: If answered “yes” to prior question, ask this) **What is the focus of this course?**  **________________________________________________** |
| **8** | **Please check all that apply, related to your background relevant to cancer biology and cancer care.** (required question)  I have academic training in cancer biology  I have been involved in conducting cancer biology research  I have been involved in conducting other cancer research  I have been involved in providing cancer patient care  None of the above |
| **9** | **Please check all that apply, related to your background relevant to drug discovery and development.** (required question)  I have academic training in drug discovery and development  I have been involved in conducting drug discovery and development research  I have been involved in business, administrative, or legal work around drug discovery or development  None of the above |

| **We are interested in learning more about course participants’ pre-course understanding of topics that will be covered in the course. Please help us by answering the following questions. The same questions will be asked at the end of the course, to assess how effective this course was in teaching this material. Your candid responses will help us improve the course for future participants.** | |
| --- | --- |
| **10** | **Please rate your current knowledge of each of the following topics in preclinical translational science. (Response options on a five-point Likert scale: No Knowledge, Slight Knowledge, Moderate Knowledge, Significant Knowledge, Expert Knowledge)** |
|  | ***GENERAL CONCEPTS*** |
|  | The difference between the terms “translational research” and “translational science” |
|  | That there are *a priori* criteria that can be used to identify promising translational research project projects |
|  | That there are scientific and operational approaches that can optimize the efficiency and effectiveness of translational research |
|  | That drug discovery and development are two different phases of a project requiring different scientific approaches and expertise |
|  | The critical importance of using multiple approaches to validate findings across all stages of the preclinical research project (e.g., high-throughput screening, target identification, toxicology testing) |
|  | ***DRUG DISCOVERY APPROACHES*** |
|  | There are different drug discovery approaches (e.g., phenotypic and target-based) that have different advantages and disadvantages |
|  | The scientific value of high-throughput screening to catalyze new directions for existing research programs |
|  | The use of multiple, rigorous target identification methods following phenotypic drug discovery approaches increases confidence that the predominant mechanism of action has been identified |
|  | That target identification following phenotypic drug discovery can generate new avenues for research |
|  | There is a need for multiple different *in vitro* assays to independently confirm a hypothesis about a biological target |

|  | ***DRUG DEVELOPMENT APPROACHES*** |
| --- | --- |
|  | The fact that medicinal chemistry approaches can be optimized to create compounds that can be used both to test a biological hypothesis and to advance the research from drug discovery to drug development |
|  | That there are limitations of individual *in vitro* and *in vivo* models when attempting to recapitulate the complexity of human disease, which can be addressed by examining data in aggregate across multiple models |
|  | That while pharmacology and toxicology testing must produce particular data to fulfill requirements for the Investigational New Drug Application to the FDA, there is nonetheless the opportunity to introduce new approaches that increase predictive accuracy and create efficiencies in how data are produced |
|  | ***CLINICAL TRIALS*** |
|  | There are principles of good clinical practice that guide the design and implementation of clinical trials |
|  | That clinical trial design and implementation must comply with guidelines from both federal and institutional oversight and regulatory bodies |
|  | ***COLLABORATIONS AND PARTNERSHIPS*** |
|  | There are particular approaches for implementing collaboration agreements and patent strategies that can facilitate effective multi-agency partnerships in translational science |
|  | There are approaches for team-based science, including approaches to communication and coordination of activities, that can maximize effective team collaboration |
| **11** | **Please indicate how strongly you agree or disagree with each of the following statements. (Response options on a five-point Likert scale: Strongly Disagree, Disagree, Undecided, Agree, Strongly Agree)** |
|  | Single investigator driven labs are typically more successful than teams of collaborators from across labs, in translational research |
|  | Teams of collaborators can accelerate the speed of translational research projects |
|  | In translational research, scientists tend to be more productive when they are working on their own research projects, compared to when they are working in teams |
|  | If you want to make important discoveries in translational research, it is important to focus any team-based work in your own discipline, versus collaborating across disciplines |
|  | While working on a research project within a particular discipline, it can be helpful to seek the perspective of other disciplines to achieve particular parts of the research aims, particularly in the context of translational research |
|  | Adding new members to your translational research team, who have varied disciplinary backgrounds, can enhance your research |
|  | Translational research teams composed of collaborators from different disciplines are more likely to fail than single-discipline teams |
|  | There are specific skills for effective cross-disciplinary collaboration in translational research, which scientists can learn |
|  | Individuals can learn team-based translational research skills at any career stage – including during their education, early-career, mid-career, and senior career |
|  | It is important to learn about the approaches, goals, and methods of other disciplines in order to know how to work together effectively in translational research projects |
| **Please complete the following brief questions, so we can learn more about our course participants.** | |
| **12** | **Which of the following best describes your work sector?** (required question)  Academia (go to question 15)  Government (go to question 13)  Industry/business/private sector (go to question 15)  Non-profit/non-governmental organization (go to question 15)  None; I am retired (go to question 15)  Other: ______________________________________ |
| **13** | (Skip pattern: If answered “Government” to prior question, ask this) **Do you currently work at the National Institutes of Health? (Please select “yes” whether you are a federal employee, contractor, or fellow)**  Yes (go to question 14)  No (go to question 15) |
| **14** | (Skip pattern: If answered “Yes” to NIH question, ask this) **At which NIH Institute or Center do you work?**  CC  CIT  CSR  FIC  NCATS  NCCIH  NCI  NCMRR  NEI  NHGRI  NHLBI  NIA  NIAAA  NIAID  NIAMS  NIBIB  NICHD  NIDA  NIDCD  NIDCR  NIDDK  NIEHS  NIGMS  NIMH  NIMHD  NINR  NINDS  NLM  OD, NIH |
| **15** | **What is the highest degree you have received to date?** (required question)  Bachelors  Masters  MD  PhD  MD/PhD  Other: please specify your highest degree here ___________________ |
| **16** | **In what discipline is your highest degree?** (required question)  Biology  Biochemistry  Chemistry  Computer Science or Informatics  Medicine  Pharmacology or Toxicology  Other: please specify the discipline of your highest degree here ___________________ |
| **17** | **How many years has it been since you received your highest degree?**  0-5 years  6-10 years  11-15 years  16-20 years  More than 20 |
| **18** | **Are you currently a fellow?**  Yes (go to question 19)  No (go to question 20) |
| **19** | *(Skip pattern: If answered “yes” to prior question, ask this)* **Which of the following best describes your fellowship?** (required question)  Post-baccalaureate Fellowship  Pre-doctoral Fellowship (currently in a doctoral training program)  Post-doctoral Fellowship (recently completed doctoral program)  Research Fellowship (fellowship that follows a post-doc, or mid-career fellowship)  Other: please specify your fellowship type here: ___________________ |
| **20** | **Are you currently enrolled as a student at a degree-granting institution (i.e., a university or college)?**  Yes (go to question 21)  No (go to closing screen) |
| **21** | (Skip pattern: If answered “yes” to Student yes/no question, ask this) **For what degree are you currently studying?** (required question)  Bachelors  Masters  PhD  MD  MD/PhD  Other: please specify the degree program here ____________________________________________ |
| **22** | (Skip pattern: If answered “yes” to Student yes/no question, ask this) **In what discipline is your current degree program?** (required question)  Biology  Biochemistry  Chemistry  Computer Science or Informatics  Medicine  Pharmacology or Toxicology  Other: please specify the discipline of your highest degree here ___________________ |

**CLOSING SCREEN:**

**Thank you very much for your time.**

**ENDPOINT SURVEY INSTRUMENT**

**Title: Help us Enhance the Course, “Principles of Preclinical Translational Science”: Post-Course Survey**

| **Q#** | **Survey Question** |
| --- | --- |
| **1** | **Do you wish to participate in the survey?** (required question)  Yes, I consent to participate (skip pattern: go to question 2)  No, I decline to participate (skip pattern: go to closing screen) |
| **Please answer the following questions about your participation in the course.** | |
| **2** | **About what percentage of the course lectures did you listen to?** (required question)  0-25%  26-50%  51-75%  76%-100% |
| **3** | **About what percentage of the required readings did you complete? Please select all that apply.** (required question)  0-25%  26-50%  51-75%  76%-100% |
| **4** | **About what percentage of the recommended readings did you complete? Please select all that apply.** (required question)  0-25%  26-50%  51-75%  76%-100% |
| **5** | **Which of the Discussion Board Reflection and Discussion Assignments did you participate in?** (required question)  None  Week 3  Week 6  Both week 3 and week 6 |
| **6** | **Which of the “Live Q and A sessions" with course speakers did you listen to? Please include both "live" and recorded sessions you listened to.** (required question)  None  Week 3  Week 6  Both week 3 and week 6 |
| **7** | **Did you submit 1 or more questions for the speakers to address during the Live Q and A sessions?**  Yes  No |
| **Please answer the following questions about the design of the course.** | |
| **8** | **How effective was the online format to teach the content?** (required question)  Not at all effective  Somewhat effective  Moderately effective  Very effective |
| **9** | **How effective was the case study approach to teach the course content?** (required question)  Not at all effective  Somewhat effective  Moderately effective  Very effective |
| **10** | **How helpful were the required readings in supplementing the lectures?** (required question)  Not at all helpful  Somewhat helpful  Moderately helpful  Very helpful |
| **11** | **How helpful were the “Live Q and A sessions”?** (required question)  Not at all helpful  Somewhat helpful  Moderately helpful  Very helpful |
| **We are interested in learning whether the course increased students’ knowledge of particular topics. Please answer the following related questions. You might notice these same questions were asked in the week 1 survey. Your candid responses will help us improve the course for future participants.** | |
| **12** | **Please rate your current knowledge of each of the following topics in preclinical translational science. (Response options on a five-point Likert scale: No Knowledge, Slight Knowledge, Moderate Knowledge, Significant Knowledge, Expert Knowledge)** |
|  | ***GENERAL CONCEPTS*** |
|  | The difference between the terms “translational research” and “translational science” |
|  | That there are *a priori* criteria that can be used to identify promising translational research project projects |
|  | That there are scientific and operational approaches that can optimize the efficiency and effectiveness of translational research |
|  | That drug discovery and development are two different phases of a project requiring different scientific approaches and expertise |
|  | The critical importance of using multiple approaches to validate findings across all stages of the preclinical research project (e.g., high-throughput screening, target identification, toxicology testing) |
|  | ***DRUG DISCOVERY APPROACHES*** |
|  | There are different drug discovery approaches (e.g., phenotypic and target-based) that have different advantages and disadvantages |
|  | The scientific value of high-throughput screening to catalyze new directions for existing research programs |
|  | The use of multiple, rigorous target identification methods following phenotypic drug discovery approaches increases confidence that the predominant mechanism of action has been identified |
|  | That target identification following phenotypic drug discovery can generate new avenues for research |
|  | There is a need for multiple different *in vitro* assays to independently confirm a hypothesis about a biological target |
|  | ***DRUG DEVELOPMENT APPROACHES*** |
|  | The fact that medicinal chemistry approaches can be optimized to create compounds that can be used both to test a biological hypothesis and to advance the research from drug discovery to drug development |
|  | That there are limitations of individual *in vitro* and *in vivo* models when attempting to recapitulate the complexity of human disease, which can be addressed by examining data in aggregate across multiple models |
|  | That while pharmacology and toxicology testing must produce particular data to fulfill requirements for the Investigational New Drug Application to the FDA, there is nonetheless the opportunity to introduce new approaches that increase predictive accuracy and create efficiencies in how data are produced |
|  | ***CLINICAL TRIALS*** |
|  | There are principles of good clinical practice that guide the design and implementation of clinical trials |
|  | That clinical trial design and implementation must comply with guidelines from both federal and institutional oversight and regulatory bodies |
|  | ***COLLABORATIONS AND PARTNERSHIPS*** |
|  | There are particular approaches for implementing collaboration agreements and patent strategies that can facilitate effective multi-agency partnerships in translational science |
|  | There are approaches for team-based science, including approaches to communication and coordination of activities, that can maximize effective team collaboration |
| **13** | **Please indicate how strongly you agree or disagree with each of the following statements. (Response options on a five-point Likert scale: Strongly Disagree, Disagree, Undecided, Agree, Strongly Agree)** |
|  | Single investigator driven labs are typically more successful than teams of collaborators from across labs, in translational research |
|  | Teams of collaborators can accelerate the speed of translational research projects |
|  | In translational research, scientists tend to be more productive when they are working on their own research projects, compared to when they are working in teams |
|  | If you want to make important discoveries in translational research, it is important to focus any team-based work in your own discipline, versus collaborating across disciplines |
|  | While working on a research project within a particular discipline, it can be helpful to seek the perspective of other disciplines to achieve particular parts of the research aims, particularly in the context of translational research |
|  | Adding new members to your translational research team, who have varied disciplinary backgrounds, can enhance your research |
|  | Translational research teams composed of collaborators from different disciplines are more likely to fail than single-discipline teams |
|  | There are specific skills for effective cross-disciplinary collaboration in translational research, which scientists can learn |
|  | Individuals can learn team-based translational research skills at any career stage – including during their education, early-career, mid-career, and senior career |
|  | It is important to learn about the approaches, goals, and methods of other disciplines in order to know how to work together effectively in translational research projects |
|  | **Please complete the following questions asking for your perspectives on the course.** |
| **14** | **Did participating in this course influence your goals for your future work? Examples might include, but are not limited to the kinds of activities you would like to engage in or your broader scientific or professional goals.** (required question)  Yes  No |
| **15** | **Why or why not? Please elaborate.** (Optional question - open ended text response) |
| **16** | **Did participating in this course influence the approaches you intend to use in your future work?** (required question)  Yes  No |
| **17** | **Why or why not? Please elaborate.** (Optional question - open ended text response) |
| **18** | **We hope to provide course participants with a unique window into the translational science process. Please rate the degree to which this course achieved this aim.** (required question)  Response options on a four-point Likert scale: Not at all, Slightly, Moderately, Completely |
| **19** | **Please rate the value of this course to you, overall.** (required question)  Response options on a four-point Likert scale: Not at all valuable, Slightly valuable, Moderately valuable, Extremely valuable |
| **20** | **Please use this space to share your thoughts on why you rated the course this way.** (optional question - open ended text response) |
| **21** | **Course participants are an ideal source of ideas for enhancing the course. Please use this space to provide your recommendations. Feel free to comment on any aspect of the course. (Examples include but are not limited to: content/topics covered, design of the course, speakers, delivery, format.)** (optional question - open ended text response) |
| **22** | **If we were to expand this course to a 2-credit offering, we would add more lectures. What current content should we elaborate on? What additional topics should we cover, that are not covered already?**(optional question - open ended text response) |
| **23** | **To help us learn more, would you be interested in elaborating on your responses in a brief interview with an NCATS Education Branch staff member?** (required question)  Yes (go to question 23)  No (go to completion screen) |
| **24** | **Please use this space to provide your name and email address. You will be contacted by a member of the NCATS Education Branch. Please Note: if you provide your name here, it will be separated from the rest of your survey responses, which are anonymized**. (optional question) |

**CLOSING SCREEN:**

**Thank you very much for your time.**
